# Effect of moderate and Severe Hypoxic exposure coupled with fatigue on psychomotor vigilance testing, muscle tissue oxygenation, and muscular performance

**DOI:** 10.1016/j.crphys.2021.11.001

**Published:** 2021-11-04

**Authors:** Cory M. Smith, Owen F. Salmon, Jasmin R. Jenkins

**Affiliations:** a Human & Environmental Physiology Laboratory, The University of Texas at El Paso, El Paso, TX, USA; b Interdisciplinary Health Sciences PhD Program, The University of Texas at El Paso, El Paso, TX, USA

**Keywords:** *Altitude*, *PVT*, *NIRS*, *TSI*, *FiO*_*2*_

## Abstract

**Purpose:**

The purpose of this study is to examine the effects of fatigue on muscular performance, oxygenation saturation, and cognition following acute hypoxic exposure at Normoxia, Moderate Hypoxia (MH), and Severe Hypoxia (SH).

**Methods:**

Twelve males performed 3 sets of leg extensions to failure under Normoxia (FiO_2_: 21%), MH (Fi0_2_: 15.4%), and SH (Fi0_2_: 12.9%). Heart rate, peripheral oxygenation saturation, total saturation index, psychomotor vigilance testing reaction time, psychomotor vigilance error rate, maximum strength, and repetitions to failure were measured throughout each visit.

**Results:**

The primary findings indicated that MH and SH resulted in significant decreases in psychomotor vigilance test performance (MH: 388.25–427.17 ms, 0.41–0.33 error rate; SH: 398.17–445.42 ms reaction time, 0.25–1.00 error rate), absolute muscle tissue oxygen saturation (Abs-StO_2_) (MH:67.22% compared to SH:57.56%), but similar muscular strength, heart rate, and patterns of muscle tissue oxygen saturation responses (StO_2_%) during fatigue when compared to Normoxia. There was an acute decrease in the ability to remain vigilant and/or respond correctly to visual stimuli as indicated by the worsened reaction time (PVT_RT_) during MH (FiO_2_: 15.4%) and increased PVT_RT_ and error rate (PVT_E_) during SH (FiO_2_: 12.9%) conditions.

**Conclusions:**

Acute hypoxic exposure in the current study was not a sufficient stimuli to elicit hypoxic-related changes in HR, muscular strength (1-RM), or repetitions to failure. The SpO_2_ responses were hypoxic-level dependent with increasing levels of hypoxia resulting in greater and more sustained reductions in SpO_2_. The combined SpO_2_ and StO_2_ responses at MH and SH suggested a balance between the muscles metabolic demand remaining lower than the muscle oxygen diffusion capacity. During the SH condition, Abs-StO_2_ suggested greater metabolic stress than Normoxia and MH conditions during the fatiguing leg extensions. The patterns of responses for StO_2_% during the three sets of leg press to failure indicated that exercise is a more potent influencer to muscle oxygenation status than hypoxic conditions (FiO_2_: 15.4 and 12.9%).

## Highlights

• Moderate Hypoxia (FiO_2_: 15.4%) coupled with fatigue resulted in a decrease in reaction time.
• Severe Hypoxia (FiO_2_: 12.9%) coupled with fatigue resulted in increased error rate and a decrease in reaction time.
• Fatigue was the primary influencer on heart rate, muscular strength, and muscular endurance and not hypoxic exposure.
• Severe Hypoxia (FiO_2_: 12.9%) resulted in greatly reduced muscle tissue oxygenation saturation before and after fatigue.

## Graphical abstract

**Figure:**
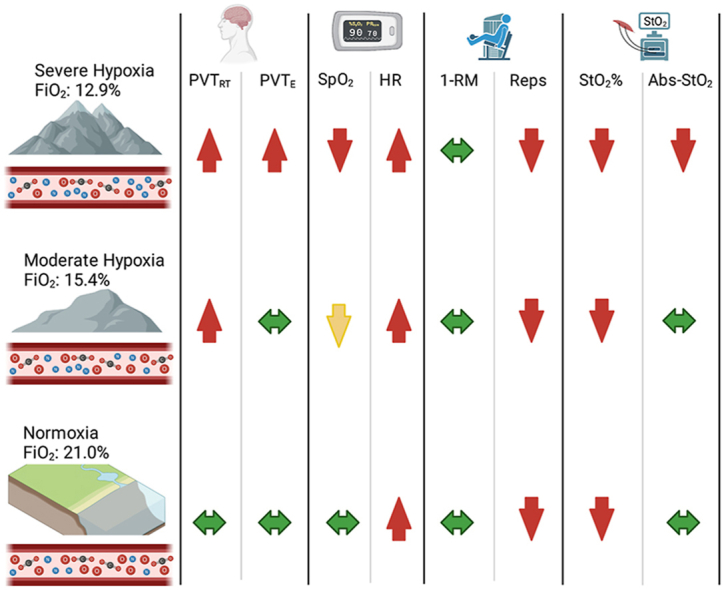

## Abbreviations:

Absolute Muscle Tissue Saturation during Recovery: StO_2_%
Differential Path-Length Factor: DPF
Fraction of Inspired Oxygen: FiO_2_
Heart Rate: HR
Moderate Hypoxia: MH
Muscle Tissue Saturation: StO_2_
Muscle Tissue Saturation during repetitions to failure: StO_2_%
One Repetition Maximum: 1-RM
Peripheral Tissue Saturation: SpO_2_
Psychomotor Vigilance Test: PVT
Psychomotor Vigilance Test Error Rate: PVT_E_
Psychomotor Vigilance Test Reaction Time: PVT_RT_
Severe Hypoxia: SH
Total Saturation Index: TSI
Vastus Lateralis: VL

## 1 Introduction

Hypoxemia can occur in low oxygen environments or low-pressure environments, which can both result in low tissue oxygen saturation, termed hypoxia (Hill et al., 2011; Lenhart, 2012). Hypoxic hypoxia is the most common type of hypoxia that occurs during mountaineering, aviation, and military activities and is due to the low-pressures resulting in fewer oxygen molecules per square inch (Lenhart, 2012). The reduction in inspired oxygen results in inefficient oxygen transfer at the lungs, ultimately causing low tissue saturation. Research examining hypoxia most frequently utilize normobaric hypoxia which achieves hypoxia through reduced FiO_2_ (McMorris et al., 2017; Millet et al., 2012; Shaw et al., 2021; Pun et al., 2018; Sander, 2016). Although there are some reported differences in the ventilatory responses between normoxic and hypobaric hypoxia, the majority of muscular and neural physiological responses as well as cognitive responses have been shown to be similar through the use of equivalent-air-altitude (McMorris et al., 2017; Shaw et al., 2021). Thus, the utilization of normobaric hypoxia can be utilized to examine the influence of hypoxia on many physiological systems within the body as well as cognitive performance under reduced blood oxygenation conditions.

The simultaneous examination of heart rate (HR), oxygen saturation (SpO_2_), and peripheral tissue oxygen saturation (StO_2_) via near-infrared spectroscopy, and cognitive function via psychomotor vigilance testing (PVT) have been shown to be useful for tracking and identifying the physiological and cognitive changes associated with fatigue and hypoxia (Basner and Dinges, 2012; Davis and Barstow, 2013; Khitrov et al., 2014; Shaw et al., 2021). Specifically, near-infrared spectroscopy allows for the measurement StO_2_ through the tissue saturation index (TSI) which is the total concentrations ratio indicating the levels of oxygen supply and oxygen consumption to the targeted muscle (Jones et al., 2016; McManus et al., 2018). A reduction in StO_2_ indicates a decrease in the localized, active muscle's oxygenation status due to reduced oxygen delivery and/or greater oxygen consumption (Jones et al., 2016; McManus et al., 2018). For example, Shannon et al. (2017) reported a fatigue-induced decrease in muscle tissue oxygenation status of the leg during a 3 km time-trial hiking exercise. Furthermore, Shannon et al. (2017) also reported greater muscle tissue oxygenation of the leg at 3000 m compared to 4300 m in healthy male subjects. Thus, tissue oxygenation metrics, such as StO_2_, through the use of near-infrared spectroscopy are sensitive to changes in oxygen availability and are capable of detecting fatigue- and dose-dependent changes in hypoxic-related tissue saturation.

The PVT has been used to examine the level of vigilant attention of those exposed to reduced sleep, hypoxia, and fatigue (Latshang et al., 2013; Oeung et al., 2020; Pun et al., 2018). Specifically, the PVT measures how quickly an individual responds to a visual stimuli as well as their accuracy in reactions to a stimuli. Thus, the PVT allows for the quantification of alertness, cognitive reaction time, and cognitive errors associated with hypoxia and fatigue (Pun et al., 2018). Determining the influence of fatigue and hypoxia on reaction time and error rate is pivotal in military personnel who often need to respond to visual threats or perform complex tasks during which errors can result in physical injury (Latshang et al., 2013; Pun et al., 2018). For example, Pun et al. (2018) reported a decrease in PVT reaction time following altitude exposure to 5050 m. Furthermore, Pun et al. (2018) suggested that the PVT performance was related to blood oxygen saturation levels, which can be negatively impacted due to fatigue (Latshang et al., 2013; Oeung et al., 2020; Pun et al., 2018).

The combination of physiological and cognitive measures during acute hypoxia exposure coupled with fatigue can provide valuable information for mission planning, task-performance expectations, and countermeasure development in mountaineering, aviation, and military activities. Therefore, the purpose of this study is to examine the effects of fatigue on muscular strength, muscular endurance, peripheral oxygenation saturation, HR, StO_2_, and PVT following acute hypoxic exposure at Normoxic (FiO_2_: 21%), Moderate Hypoxia (MH) (Fi0_2_: 15.4%; simulated 2438 m), and Severe Hypoxia (SH) (FiO_2_: 12.9%; simulated 3810 m). It was hypothesized a fatigue- and hypoxic-induced decrease in muscular strength, muscular endurance, SpO_2_, StO_2_, and PVT scores with a concomitant increase in HR (Lenhart, 2012; Pun et al., 2018; Shannon et al., 2017).

## 2 Material and methods

### 2.1 Participants

Twelve males participated in the study (age: 20.9 ± 3.4 yr, height: 175.3 ± 11.2 cm, weight: 85.6 ± 14.3 kg). All subjects lived at ∼1,000m without any extended travel (greater than 24-hr) to altitudes greater than 1524 m within the past 6-mo. In addition, all subjects were trained (minimum 3 days per week for 1-hr a day consistently for at least 6-mo) and free from any musculoskeletal injuries or neuromuscular disorders. Subjects were non-smokers, free from any asthma, or history of acute mountain sickness and asked to refrain from consuming any caffeine or alcohol within 24-hr of their scheduled testing visit. Subjects were instructed to maintain a similar diet throughout the duration of the study. Subjects completed an informed consent and health history questionnaire prior to participation in the study. A F-test repeated measures ANOVA a priori power analysis was performed from the psychomotor vigilance time data of Pun et al. (2018) which indicated an n = 9 was needed to reach a power of 0.80. The study was approved by the University's Institutional Ethical Review Board (approval no. 1482628–1) and complied with standards set in the Declaration of Helsinki (WMA, 2013). During the consent process, subjects were informed about the purpose, testing procedures, and risks associated with those procedures prior to agreeing to participate in this study.

### 2.2 Study design

The study utilized a repeated measures, cross-over design with the order of hypoxic exposure being randomized for each subject. The study consisted of four visits to the laboratory and all visits occurred at the same time of day ±1-hr. The first visit familiarized the subjects to all testing procedures, including acute hypoxic exposure and assessments including StO_2_ on the vastus lateralis (VL), HR, SpO_2_, and PVT. After subjects were familiarized with the testing procedures, all follow-up visits were scheduled.

Visits 2, 3, and 4 consisted of identical testing protocols with the level of hypoxia being randomized at MH (FiO_2_: 15.4%; simulated 2438 m) or SH (FiO_2_: 12.9%; simulated 3810 m). The Normoxic (FiO_2_: 21%) visit for all subjects was performed first to normalize one repetition maximum (1-RM) and repetition to failure resistance for the MH and SH visits. All FiO_2_ was blinded to subjects throughout each visit. During the beginning of each visit, subjects were instructed to sit in a chair for 10-min to obtain resting StO_2_, HR, SpO_2_, and PVT performance as Pre-Exposure measures. Subjects were then exposed to that visits hypoxic exposure level (Normoxic, MH, or SH) for 30-min while sitting relaxed in a chair. Following the initial 30-min hypoxic exposure period, Pre-Fatigue measures for StO_2_, HR, SpO_2_, and PVT performance were obtained prior to the fatiguing leg extension tasks. Following the collection of the Pre-Fatigue measures, a structured warm-up and leg extension 1-RM procedure of the dominant leg (based on kicking preference) was performed in accordance with the National Strength and Conditioning Associations protocol under the supervision of a Certified Strength and Conditioning Specialist (Baechle et al., 2008). After obtaining the leg extension 1-RM, a 3-min rest was provided and verified StO_2_, HR, and SpO_2_ returned to pre 1-RM values before beginning the fatiguing leg extension protocol.

### 2.3 Fatiguing protocol

The fatiguing leg extension protocol consisted of 3 sets of leg extensions to failure at 70% 1-RM based on Normoxic (FiO_2_: 21%) visits 1-RM with 1-min of rest between each set. Immediately after the 3 sets of leg extensions to failure, StO_2_, PVT, HR, and SpO_2_ were measured. During the 3 sets of repetitions to failure, muscle tissue oxygenation saturation during percent of repetition (StO_2_%) data from the VL was collected during the repetition that corresponded to initial, 50, and 100% of the repetitions to failure. Furthermore, absolute recovery muscle tissue oxygenation saturation (Abs-StO_2_) was measured immediately before each set of leg extensions.

### 2.4 Hypoxia

Hypoxia was induced using a Hypoxico HYP123 altitude generator (Hypoxico, New York, NY USA) with an in-line 300L Douglas bag with a pressure relief valve connected to a Hans-Rudolph 7450 full face metabolic mask (Shawnee, KS USA). A calibrated in-line MySign 0 sensor was utilized to monitor the oxygen percent being delivered to the subjects (FiO_2_) under normobaric hypoxia conditions (Envitec Wismar, Germany). During all testing, humidity, pressure, and temperature were controlled for each visit at 20 °C and 35% relative humidity as well as to align with effective-air-altitude equivalencies.

### 2.5 One-Repetition maximum

The 1-RM and repetition to failure leg extensions were performed on a commercial Cybex leg extension machine with an attached weight stack (Life Fitness, Rosemont, IL USA). Range of motion was controlled and monitored throughout testing. Failure was determined if subjects were unable to perform a full range of motion during the 1-RM or repetitions to failure. All 1-RM procedures were performed by Certified Strength and Conditioning Specialists and in accordance with the National Strength and Conditioning Associations testing procedures (Baechle et al., 2008). Specifically, the subjects performed a warm-up set of 5–10 repetitions at approximately 50% of an estimated 1-RM, and then 3 to 5 repetitions at approximately 75% of their estimated 1-RM. A 1-min rest was given between trials. The subjects then performed a series of single repetitions to determine their 1-RM leg extension within 2.27 kg. A 1-RM was performed before each testing visit, however, the calculated 70% 1-RM weight for all repetitions to failure were based on the 1-RM from the Normoxic visit. During the repetitions to failure, subjects performed as many repetitions as possible until either they could lift the weight through a full range of motion or they felt that they can no longer perform any additional leg extensions. Failure was also determined if subjects paused for greater than 1-s between repetitions. The leg extension was chosen as it has been reported to track military task-performance such as navigating obstacles and moving heavy loads while reducing confounding metabolic influences of full body movements (Alvar et al., 2017; Bishop et al., 1999; Lenhart, 2012; Scofield and Kardouni, 2015).

### 2.6 Heart rate & SpO_2_

Heart rate and SpO_2_ were measured using a vital sign monitor before exposure to hypoxia, pre-fatigue (before performing leg extension muscle actions), and post-fatigue (immediately after completing the third set of repetitions to failure). Heart rate is resented at beats per minute. The SpO_2_ was taken peripherally at the index finger of the non-dominant hand and averaged over a 10-s period. This allowed for simultaneous measurements of the SpO_2_ while leaving the dominant hand available to perform the PVT.

### 2.7 Psychomotor vigilance test

The 3-min PVT was performed on a touch screen tablet with standard hand placements and distance from the subject (Apple Cupertino, CA USA). The subjects rested their hand on a set location at the bottom right corner of the device and when the number appeared, they immediately would tap the number on the center of the screen (Basner and Dinges, 2012; Khitrov et al., 2014; Matsangas et al., 2017; Pun et al., 2018; Rajaraman et al., 2012). The time from when the numbers appeared on the screen until the subjects response was recorded as PVT_RT_ and the number of errors were recorded as PVT_E_. An error was recorded if subjects tapped the screen when there was not a number displayed. The PVT test was performed pre-exposure, pre-fatigue, and post-fatigue.

### 2.8 Muscle tissue oxygenation

All muscle tissue oxygenation saturation values, StO_2_, of the dominant legs VL was measured using TSI with a near-infrared spectroscopy device (Artinis Medical Systems Einsteinwig, Nethlerlands). A single channel, dual optode setup was utilized with an average optode to receiver distance of 45 mm. Each optode consisted of a dual wavelength of 848 and 762 nm. A differential path-length factor (DPF) of 4.0 was utilized for all measurements. The signal was sampled at 10 Hz for all subjects. In addition, the signals amplification and power were determined for each subject during the familiarization visit and were held constant for each subsequent visit. This allowed for repeatability of the measurement and improved the sensitivity signals. The StO_2_ was measured before each set (Abs-StO_2_), and during the repetitions to failure (StO_2_%). During the repetitions to failure, only the repetitions corresponding to Initial, 50, and 100% of repetitions to failure were used for analysis. The Initial, 50, and 100% of the repetitions to failure were utilized to allow subjects data to be compared as a function of volitional exhaustion being the indicator of fatigue. The StO_2_% for the repetitions to failure were normalized to the initial repetition of the control set which reflects the pattern of responses for muscle oxygenation during each set compared to Normoxic conditions. The Abs-StO_2_ reflects the level of muscle oxygenation prior to each set which reflects the initial muscle oxygenation prior to each set.

### 2.9 Statistical analyses

A one-way repeated measures ANOVA was performed on the 1-RM strength across Hypoxic visits (Normoxic, MH, and SH) to examine if acute hypoxic exposure altered maximal strength. In addition intra class correlation coefficients (ICC) were determined to examine reliability using Model 2,1 via SPSS (ICC_2,1_) from the Normoxia, MH, and SH data at the Pre-Exposure time point for SpO_2_ (ICC_2,1_: 0.657), HR (ICC_2,1_: 0.497), and PVT_RT_ (ICC_2,1_: 0.797) as well as the 1-RM trials (ICC_2,1_: 0.949). Two separate, 3 (Hypoxia: Normoxic, MH, and SH) x 3 (Repetitions: Set 1, Set 2, and Set 3) two-way repeated measures ANOVAs were performed on the number of repetitions to failure performed and Abs-StO_2_. In addition, four separate, 3 (Hypoxia: Normoxic, MH, and SH) x 3 (Time: Pre-Exposure, Pre-Fatigue, and Post-Fatigue) two-way repeated measures ANOVAs were performed on HR, SpO_2_, PVT_RT_, and PVT_E_. A 3 (Hypoxia: Normoxic, MH, and SH) x 3 (Set: Set 1, Set 2, and Set 3) x 3 (Repetitions: Initial, 50%, and 100% repetitions to failure) three-way repeated measures ANOVAs was performed on the StO_2_% normalized to initial repetition. Follow-up two- and one-way ANOVAs as well as post-hoc paired sampled *t*-tests with Tukey-LSD were performed when appropriate. If sphericity was violated during any ANOVA, the Greenhouse-Geisser correction was used. An alpha of p ≤ 0.05 was considered statistically significant for all statistical analyses (IBM SPSS Version 25.0, Armonk, NY).

## 3 Results

### 3.1 PVT_RT_

**Fig. 1:**
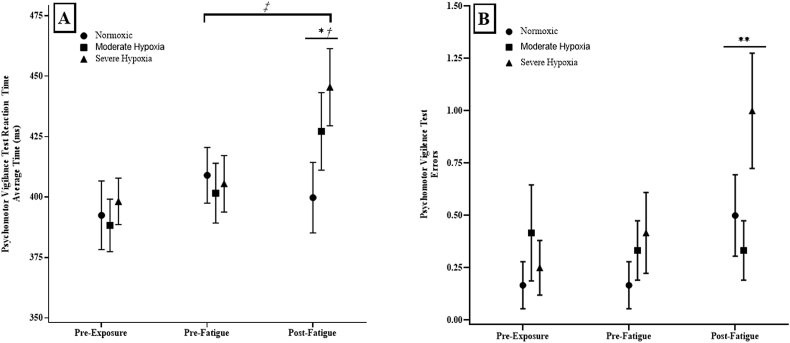
**A)** Psychomotor vigilance test average reaction time (PVT_RT_) and **B)** Psychomotor vigilance test average errors (PVT_E_) at Pre-Exposure, Pre-Fatigue, and Post-Fatigue at simulated Normoxic, Moderate Hypoxia, and Severe Hypoxia. *: Denotes Moderate Hypoxic and Severe Hypoxic were significantly greater than Normoxic. **: Indicates that Severe Hypoxia condition had a significantly greater PVT_E_ at Post-Fatigue compared to Pre-Exposure and Pre-Fatigue. †: Indicates that Pre-Exposure was significantly less than Post-Fatigue for Moderate Hypoxia and Severe Hypoxia. ‡: Indicates that Pre-Fatigue was significantly less than Post-Fatigue for Moderate Hypoxia and Severe Hypoxia.

There was a significant 2-way (Hypoxia x Time) interaction (p = 0.028; η$\begin{array}{l} 2\\ p \end{array}$ = 0.214) for the repeated measures ANOVA from the PVT_RT_. The follow-up one-way repeated measures ANOVA across Time for Normoxic indicated no significant difference between PVT_RT_ (p = 0.371; η$\begin{array}{l} 2\\ p \end{array}$ = 0.082). The one-way repeated measures ANOVA across Time for MH was significant (p < 0.01; η$\begin{array}{l} 2\\ p \end{array}$ = 0.48) which indicated that Pre-Exposure = Pre-Fatigue (p = 0.081), Pre-Fatigue < Post-Fatigue (p = 0.027), and Pre-Exposure < Post-Fatigue (p < 0.01). In addition, the one-way repeated measures ANOVA across time for SH was significant (p = 0.01; η$\begin{array}{l} 2\\ p \end{array}$ = 0.368) which indicated that Pre-Exposure = Pre-Fatigue (p = 0.438), Pre-Exposure < Post-Fatigue (p = 0.010), and Pre-Fatigue < Post-Fatigue (p = 0.031). Furthermore, there was a significant one-way repeated measures ANOVA across hypoxia for PVT_RT_ (p < 0.001; η$\begin{array}{l} 2\\ p \end{array}$ = 0.731) which indicated that Normoxic < MH (p < 0.01), MH = SH (p < 0.05), and Normoxic < SH (p < 0.01) (Fig. 1A).

### 3.2 PVT_E_

There was a significant 2-way interaction (p = 0.021; η$\begin{array}{l} 2\\ p \end{array}$ = 0.226) for the repeated measures ANOVA from the PVT_E_. The follow-up one-way repeated measures ANOVA across time for Normoxic indicated no significant difference between PVT_E_ (p = 0.197; η$\begin{array}{l} 2\\ p \end{array}$ = 0.211). The follow-up one-way repeated measures ANOVA across time for MH indicated no significant difference between PVT_E_ (p = 0.355; η$\begin{array}{l} 2\\ p \end{array}$ = 0.654). The one-way repeated measures ANOVA across time for SH, however, was significant (p < 0.01; η$\begin{array}{l} 2\\ p \end{array}$ = 0.390) which indicated that Pre-Exposure = Pre-Fatigue (p = 0.439), Pre-Exposure < Post-Fatigue (p = 0.012), and Pre-Fatigue < Post-Fatigue (p = 0.001). The one-way repeated measures ANOVAs across hypoxia for time were not significant for Pre-Exposure (p = 0.264; η$\begin{array}{l} 2\\ p \end{array}$ = 0.114) or Pre-Fatigue (p = 0.342; η$\begin{array}{l} 2\\ p \end{array}$ = 0.083), but was for Post-Fatigue (p = 0.043; η$\begin{array}{l} 2\\ p \end{array}$ = 0.292) which indicated that Normoxic = MH (p = 0.080), Normoxic < SH (p = 0.048), and MH < SH (p = 0.032) (Fig. 1B).

### 3.3 SpO_2_

**Fig. 2:**
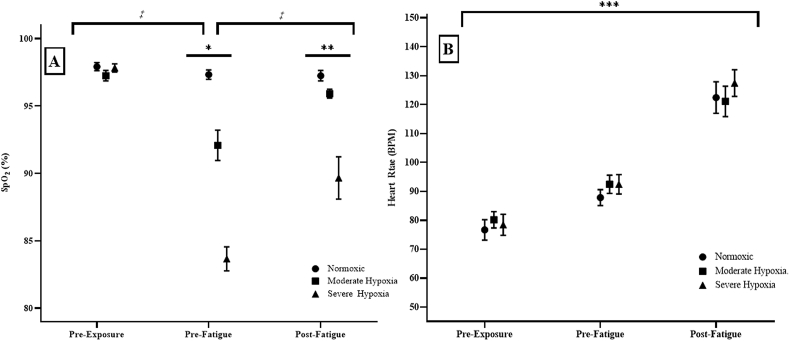
**A)** Peripheral oxygenation saturation (SpO_2_) and **B)** heart rate (HR; beats per minutes; BPM) for Pre-Exposure, Pre-Fatigue, and Post-Fatigue at simulated Normoxic, Moderate Hypoxia, and Severe Hypoxia. *: Indicates that Moderate Hypoxia and Severe Hypoxia conditions decreased significantly from Pre-Exposure to Pre-Fatigue. **: Indicates the Severe Hypoxia condition decreased significantly from Pre-Exposure to Post-Fatigue. ***: Indicates that Pre-Exposure was significantly less than Pre-Fatigue, and that Pre-Fatigue was significantly less than Post-Fatigue for all conditions. ‡: Indicates that Normoxic was significantly greater than Moderate Hypoxia and Severe Hypoxia at Pre-Fatigue and Post-Fatigue. In addition, Moderate Hypoxia was significantly greater than Severe Hypoxia at Pre-Fatigue and Post-Fatigue.

There was a significant 2-way interaction (p < 0.001; η$\begin{array}{l} 2\\ p \end{array}$ = 0.742) for the repeated measures ANOVA from the SpO_2_. The one-way repeated measures ANOVA across time for MH was significant (p < 0.01; η$\begin{array}{l} 2\\ p \end{array}$ = 0.597) which indicated that Pre-Exposure > Pre-Fatigue (p < 0.001), Pre-Fatigue < Post-Fatigue (p < 0.001), and Pre-Exposure = Post-Fatigue (p < 0.01). In addition, the one-way repeated measures ANOVA across time for SH was significant (p = 0.01; η$\begin{array}{l} 2\\ p \end{array}$ = 0.721) which indicated that Pre-Exposure > Pre-Fatigue (p < 0.01), Pre-Exposure > Post-Fatigue (p < 0.001), and Pre-Fatigue < Post-Fatigue (p < 0.001). The one-way repeated measures ANOVAs across hypoxia for time were significant Pre-Fatigue (p < 0.01; η$\begin{array}{l} 2\\ p \end{array}$ = 0.882) and Post-Fatigue (p < 0.001; η$\begin{array}{l} 2\\ p \end{array}$ = 0.661) which both indicated that Normoxic > MH (p < 0.01), MH > SH (p < 0.01), and Normoxic > SH (p < 0.01) (Fig. 2A).

### 3.4 Heart rate

There was no significant two-way repeated measures ANOVA (p = 0.423; η$\begin{array}{l} 2\\ p \end{array}$ = 0.085) or main effect (p = 0.569; η$\begin{array}{l} 2\\ p \end{array}$ = 0.045) for HR. There was, however, a main effect for time (p < 0.01; η$\begin{array}{l} 2\\ p \end{array}$ = 0.943) which indicated that Pre-Exposure < Pre-Fatigue (p < 0.01), Pre-Fatigue < Post-Fatigue (p < 0.01), and Pre-Exposure < Post-Fatigue (p < 0.01) (Fig. 2B).

### 3.5 Repetition maximum

**Fig. 3:**
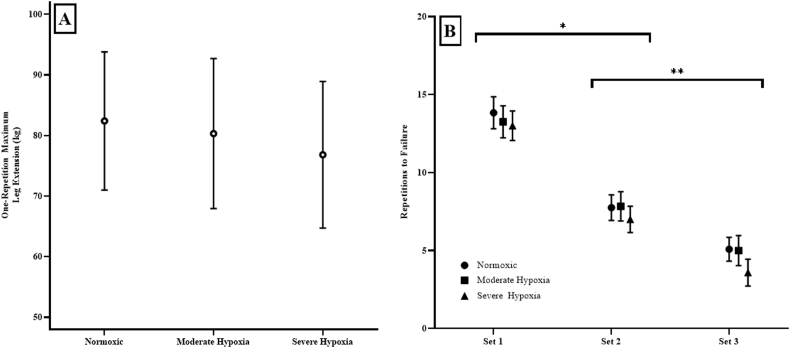
**A)** One-Repetition maximum (1-RM) leg extension in kg and **B)** Leg extension repetitions to failure at 70% 1-RM for Pre-Exposure, Pre-Fatigue, and Post-Fatigue collapsed across simulated Normoxic, Moderate Hypoxia, and Severe Hypoxia. There was no significant differences in 1-RM leg extension weight across conditions. *: Indicates that Set 1 was significantly greater than Set 2 for all conditions. **: Indicates that Set 2 was significantly greater than Set 3 for all conditions.

For 1-RM strength, the one-way repeated measures ANOVA was not significant across hypoxic exposure (p = 0.105; η$\begin{array}{l} 2\\ p \end{array}$ = 0.121) (Fig. 3A).

### 3.6 Repetitions to failure

There was a significant 2-way interaction (p = 0.047; η$\begin{array}{l} 2\\ p \end{array}$ = 0.170) for the repeated measures ANOVA from the number of repetitions to failure. The follow-up one-way repeated measures ANOVAs across time for Normoxic (p < 0.01; η$\begin{array}{l} 2\\ p \end{array}$ = 0.904), MH (p < 0.01; η$\begin{array}{l} 2\\ p \end{array}$ = 0.804), and SH (p < 0.01; η$\begin{array}{l} 2\\ p \end{array}$ = 0.844) were significant which indicated that Set 1 > Set 2 (p < 0.01), Set 2 > Set 3 (p < 0.01), and Set 1 > Set 3 (p < 0.01). The one-way repeated measures ANOVA across hypoxia for Set 1 (p = 0.389; η$\begin{array}{l} 2\\ p \end{array}$ = 0.082), Set 2 (p = 0.616; η$\begin{array}{l} 2\\ p \end{array}$ = 0.043), and Set 3 (p = 0.270; η$\begin{array}{l} 2\\ p \end{array}$ = 0.112) were not significant (Fig. 3B).

### 3.7 Abs-StO_2_

**Fig. 4:**
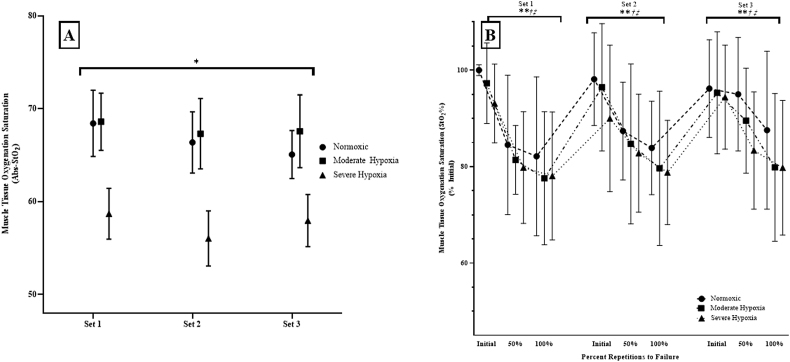
**A)** Absolute muscle tissue oxygenation saturation (Abs-StO_2_) and for Pre-Exposure, Pre-Fatigue, and Post-Fatigue at Normoxic, Moderate Hypoxia, and Severe Hypoxia as well as **B)** normalized StO_2_% at Initial, 50%, and 100% of repetitions to failure during Set 1, Set 2, and Set 3. *: Indicates that Severe Hypoxia Abs-StO _2_ was lower than Normoxic and Moderate Hypoxia Abs-StO_2_ at Set 1, Set 2, and Set 3. **: Indicates that the Initial repetition is significantly greater than 50% repetitions and that 50% was significantly greater than 100% of repetitions to failure. †: Indicated that for the 50% StO _2_% repetitions to failure values, Set 1 was significantly greater than Set 2 and Set 2 was significantly greater than Set 3. ‡: Indicated that for the 100% StO _2_% repetitions to failure values, Set 3 was significantly less than Set 1 and Set 2.

For the Abs-StO_2_, there was no significant Set 2-way interaction (p = 0.568; 0.055) or main effect for Set (p = 0.281; η$\begin{array}{l} 2\\ p \end{array}$ = 0.107). There was, however, a significant main effect hypoxia for the Abs-StO_2_ (p = 0.029; η$\begin{array}{l} 2\\ p \end{array}$ = 0.279) which indicated that Normoxia = MH (p = 0.617), MH > SH (p < 0.01), and Normoxic > SH (p < 0.01) (Fig. 4A).

### 3.8 StO_2_%

For the StO_2_% during repetitions to failure (normalized to the initial repetition of the control set), there was no significant 3-way repeated measures ANOVA (p = 0.249; η$\begin{array}{l} 2\\ p \end{array}$ = 0.239) or 2-way interaction for Hypoxia x Set (p = 0.793; η$\begin{array}{l} 2\\ p \end{array}$ = 0.057). The follow-up one-way repeated measures ANOVAs across repetitions for Set 1 (p < 0.01; η$\begin{array}{l} 2\\ p \end{array}$ = 0.492), Set 2 (p < 0.01; η$\begin{array}{l} 2\\ p \end{array}$ = 0.426), and Set 3 (p < 0.01; η$\begin{array}{l} 2\\ p \end{array}$ = 0.301) were significant which indicated that Initial >50% (p < 0.01), 50% > 100% (p < 0.01), and Initial >100% (p < 0.01).

The follow-up one-way repeated measures ANOVA across Sets for repetition were not significant for the Initial repetition (p = 0.289; 0.036), but were significant for 50% and 100% of the StO_2_% during the repetitions to failure. For the StO_2_% during the 50% of repetitions to failure, Set 1 > Set 2 (p < 0.01), Set 2 > Set 3 (p = 0.028), and Set 1 > Set 3 (p < 0.01). For the StO_2_% during the 100% of repetitions to failure, Set 1 = Set 2 (p = 0.329), Set 2 < Set 3 (p = 0.012), and Set 1 < Set 3 (p = 0.017). In addition, there was a main effect for repetition (p < 0.01; η$\begin{array}{l} 2\\ p \end{array}$ = 0.621) which indicated that Initial >50% (p < 0.01) < 100% (p < 0.01), and Initial <100% (p < 0.01) (Fig. 4B).

## 4 Discussion

The primary findings of this study indicated that acute MH and SH exposure resulted in significant differences in PVT_T_, PVT_E_, SpO_2_, and Abs-StO_2_, but similar 1-RM strength, HR, and patterns of StO_2_% during high-intensity fatigue when compared to Normoxia. Specifically, PVT_T_ was greater in both the MH and SH conditions compared to the Normoxia and were similar from Pre-Exposure to Pre-Fatigue (Fig. 1A and B). Although there were no differences in the increased PVT_T_ between the MH and SH conditions, there was a greater PVT_E_ during the SH compared to the Normoxic and MH conditions (Fig. 1B). Unlike previous studies which suggested that SpO_2_ tracks cognition changes, we did not observe this pattern as the lowest measured SpO_2_ values occurred at Pre-Fatigue with an increase in SpO_2_ at Post-Fatigue (Fig. 2A) (Pun et al., 2018; McMorris et al., 2017). It is plausible that the differences between our study and those who have reported SpO_2_ tracking PVT_T_ and PVT_E_ are related to how the mode of exercise effects cerebral blood flow (Ogoh and Ainslie, 2009). That is, the majority of studies which have conducted acute hypoxic exposure in conjunction with SpO_2_ measurements have been during aerobic tasks, such as mountaineering (Pun et al., 2018; McMorris et al., 2017). The present study, however, utilized acute hypoxic exposure while performing high-intensity strength movements aimed at tracking military task-performance such as navigating obstacles, breaching, and moving heavy loads such as barricades, ammo, soldiers, or weaponry (Bishop et al., 1999; Lenhart, 2012; Alvar et al., 2017; Scofield and Kardouni, 2015). Ogoh & Ainslie (2009) indicated that aerobic exercise, up to 60% maximal oxygen uptake, results in an elevated cerebral blood flow while heavy exercises has been shown to elicit reduced cerebral blood flow. Since the brain consumes a large amount of oxygen, the shunting of blood away from the brain to the body during heavy exercises, such as in the current study, may partially explain the decrease in PVT_T_ with increased SpO_2_ at Post-Fatigue compared to Pre-Fatigued values during the MH and SH conditions.

### 4.1 Psychomotor vigilance test

Reaction time, PVT_RT_, was greater in the MH and SH conditions compared to the Normoxic condition. There was no difference in PVT_RT_ between the MH and SH conditions (Fig. 1A). These findings were in agreement with those of Pun et al. (2018) who reported an ∼26% increase in PVT_RT_ following acute exposure to 2590 m, however, PVT_RT_ returned to baseline following 6-d of altitude exposure. In addition, Pun et al. (2018) reported that PVT_RT_ was highly correlated to acute mountain sickness symptoms (r = 0.62) which suggested that acute hypoxia reduces cognitive processing speed and/or movement during vigilance tasks increasing the time to complete the task. It has been hypothesized that the magnitude and duration of hypoxia greatly influences cognitive and vigilance outcomes (Pun et al., 2018; Shaw et al., 2021).

The findings of the current study support the hypotheses of the magnitude of hypoxia results in worsening cognition and vigilance (McMorris et al., 2017; Pun et al., 2018; Shaw et al., 2021). Specifically, in the current study, MH resulted in greater PVT_RT_ while SH exhibited greater PVT_RT_ and PVT_E_ compared to the Normoxia condition. The similar PVT_RT_ between the MH and SH suggested that an increase hypoxic exposure did not reduce the processing time, however, as the severity of hypoxia increases the error rate associated with vigilance detection is greatly increased (Fig. 1). These results are similar to the hypoxic-induced decreases in cognitive performance reported by McMorris et al. (2017), which attributed these decreases to hypoxic-induced reductions in the turnover rates of dopamine, norepinephrine and 5-hydroxytryptamine (Decamp and Schneider, 2009; Kumar et al., 2011; McMorris et al., 2017; Shukitt-Hale et al., 1998). These decreased neurotransmitters turnover rates result in a similar physiological and neuro-cognitive response leading to poor levels of excitations and, ultimately, reduced vigilance performance (Decamp and Schneider, 2009; Kumar et al., 2011; McMorris et al., 2017; Shukitt-Hale et al., 1998). Thus, the increased PVT_RT_ during MH and increased PVT_RT_ and PVT_E_ during SH indicates an acute decrease in the ability to remain vigilant and respond correctly to visual stimuli, which may be attributed to a reduction in neurotransmitter turnover rate.

### 4.2 SpO_2_ and heart rate

Unlike previous studies (McMorris et al., 2017; Pun et al., 2018) which suggested that SpO_2_ tracks changes in cognition, we did not observe this pattern as the lowest measured SpO_2_ values occurred at Pre-Fatigue with an increase in SpO_2_ at Post-Fatigue (Fig. 2A). It is plausible that the differences between our study and those who have reported SpO_2_ tracking PVT_RT_ and PVT_E_ are related to how the mode of exercise effects cerebral blood flow (Ogoh and Ainslie, 2009). That is, the majority of studies which have conducted acute hypoxic exposure in conjunction with SpO_2_ measurements have been during aerobic tasks, such as mountaineering (McMorris et al., 2017; Pun et al., 2018). The present study, however, utilized acute hypoxic exposure while performing high-intensity strength movements aimed at tracking military task-performance such as navigating obstacles, breaching, and moving heavy loads such as barricades, ammo, soldiers, or weaponry (Alvar et al., 2017; Bishop et al., 1999; Lenhart, 2012; Scofield and Kardouni, 2015). Ogoh and Ainslie (2009) indicated that aerobic exercise (≤60% maximal oxygen uptake) results in an elevated cerebral blood flow while heavy/high intensity exercises elicit a reduction in cerebral blood flow. Since the brain consumes a large amount of oxygen, the relative shunting of blood away from the brain to the body during heavy exercises, such as in the current study, may partially explain the decrease in PVT_RT_ with increased SpO_2_ at Post-Fatigue compared to Pre-Fatigued values during the MH and SH conditions.

Heart rate exhibited similar responses across all conditions at Pre-Exposure, Pre-Fatigue, and Post-Fatigue, which was contrary to our hypothesis (Fig. 2B). The majority of literature has shown an increased resting and exercise HR under hypoxic conditions as a result of greater sympathetic nervous system activation releasing greater amounts of epinephrine and norepinephrine (Attias et al., 2017; Koller et al., 1988; Mazzeo, 2008). For example, Sander (2016) indicated that acute altitude exposure increased HR and sympathetic activity throughout the first 24-hr of exposure and was reduced back to baseline after 10-days (4100–5260 m). In addition, the similar HR response, but generally lower SpO_2_ during MH and SH conditions suggested that high-intensity exercise was a greater mediator to HR than hypoxemia status. The majority of research has examined aerobic or long exposure durations with little emphasis placed on high-intensity exercise physiological responses (McMorris et al., 2017; Pun et al., 2018; Sanders, 2016; Shaw et al., 2021). The majority of studies interventions were aerobic and longer in duration which target different physiological energy systems, neuromuscular responses, as well as a reduced reliant on hypoxemia status for the regulation of HR than the present study (Guo et al., 2010; Hill et al., 2011; Lucía et al., 1999; Taylor and Bronks, 1994). Additional research would be needed to further identify the mechanisms resulting in high-intensity exercise regulating HR responses greater than hypoxemia as previously reported. Thus, given the findings of the current study, it is likely that acute hypoxic exposure while performing repeated, short duration, high intensity exercise may not have provided sufficient stimulus to elicit hypoxic-specific changes in HR responses. Furthermore, this study indicated that acute hypoxic exposure did not influence muscular strength or muscular endurance measured by 1-RM and repetitions to failure, respectively (Fig. 2, Fig. 3B).

### 4.3 Abs-StO_2_: recovery muscle tissue oxygen saturation

The Abs-StO_2_ indicated that Normoxic and MH Conditions muscle tissue oxygenation recovery status was not influenced by exercise or the combined effects of moderate levels of hypoxia (FiO_2_: 15.4%) and exercise. The similar Abs-StO_2_ for Normoxia and MH indicate that 1-min of recovery following the fatiguing leg extensions did not influence Abs-StO_2_ from the VL. It is likely that the maintenance of muscle tissue saturation when recovering was due to an increase in oxygenated blood flow to the active muscle and sufficient diffusive and convective processes (Bourdillon et al., 2009). For example, Bourdillon et al. (2009) indicated that low-level hypoxia coupled with exercise may decrease systemic oxygenation (SpO_2_), but not influence muscle tissue oxygenation diffusion status resulting in sufficiently oxygenated myoglobin at the active muscle. In addition, according to Shibuya and Tanaka (2003), StO_2_ is highly dependent on muscle oxygen diffusion capacity and that StO_2_ is intensity-specific with an increase in intensity resulting in a decrease in StO_2_. Thus, the reduced oxygen availability during the MH condition resulted in a decrease in SpO_2_ at Pre-Fatigue while Abs-StO_2_ at the same time-point remained unchanged which may be attributed to the balance between the muscles metabolic demand remaining lower than the muscle oxygen diffusion capacity despite being under MH (Shaw et al., 2021; Shibuya and Tanaka, 2003).

Unlike Normoxic and MH Conditions, SH exhibited lower Abs-StO_2_ across all Sets indicating that the SH (FiO_2_: 12.9%) resulted in a decrease in oxygenation status at the muscle tissue (Abs-StO_2_) and systemic oxygenation (SpO_2_). The lower Abs-StO_2_ and SpO_2_ at SH suggested a reduction in hemoglobin and myoglobin saturation throughout the duration of the SH condition (Fig. 4A and B) (Davis and Barstow, 2013). Near-Infrared Spectroscopy cannot distinguish the difference between hemoglobin and myoglobin saturation, however, it is influenced by both when measuring StO_2_ (Davis and Barstow, 2013). For example, Davis and Barstow (2013) indicated that myoglobin contributes approximately 50+% of the StO_2_ measurement when calculated from NIRS devices during exercise. In addition, Spires et al. (2011) indicated that hemoglobin and myoglobin responses can differ depending on the physiological and pathophysiological conditions employed throughout testing. Specifically, exercise intensity, duration, and oxygen availability (i.e. hypoxia) result in greater reductions in myoglobin oxygenation and are tracked within the NIRS StO_2_ signal (Davis and Barstow, 2013; Spires et al., 2011). It is important to note that Spires et al. (2011) simultaneously measured hemoglobin and myoglobin in conjunction with NIRS StO_2_ measurements, however, StO_2_ is currently unable to distinguish between the individual contributions from hemoglobin and myoglobin to NIRS derived StO_2_ values. Thus, it is plausible that the sustained decrease in Abs-StO_2_ accompanied by an increase in SpO_2_ at Post-Fatigue (although not to Pre-Exposure levels) following the sets of leg extension to failure during SH were due to greater myoglobin extraction (Davis and Barstow, 2013; Spires et al., 2011). It has been suggested that the greater reduction in myoglobin saturation may have occurred because of increased energy expenditure due to the reduced oxygen availability placing greater metabolic stress on the localized muscle during SH (Bourdillon et al., 2009; Davis and Barstow, 2013; Shaw et al., 2021; Shibuya and Tanaka, 2003). Therefore, during the SH condition, the localized muscle likely experienced greater metabolic stress during the fatiguing leg extension tasks resulting in lower muscle oxygenation levels indicating a greater systemic prioritization placed on oxygenation.

### 4.4 StO_2_%: fatiguing muscle tissue oxygen saturation

The StO_2_% responses during the three sets of leg extension muscle actions to failure at 70% 1-RM were similar across Normoxic, MH, and SH conditions with a decrease from Initial at 50 and 100% of the repetitions to failure (Fig. 4B). These findings were similar to those of Millet et al. (2009) who reported similar StO_2_% during fatiguing, submaximal isometric forearm flexion movements at normoxic and hypoxic conditions (FiO_2_: 9 and 14%). The fatigue-induced decrease in StO_2_% at 50 and 100% of the repetitions to failure during all three sets indicated that the metabolic demand required similar oxygen requirements despite differences in oxygen availability. It has been hypothesized that the muscular oxygen demand during exercise are influenced greater by the diffusion capacity and less by the arterial oxygen saturation status. The findings the current and previous studies (Davis and Barstow, 2013; Millet et al., 2009, 2012; Shaw et al., 2021) suggested that during exercise, extraction oxygen in the active muscle is a more potent influencer to NIRS derived StO_2_% metrics during exercise than hypoxic exposure. Thus, these findings indicated that exercise is a more potent influencer to muscle oxygenation status than hypoxic conditions (FiO_2_: 15.4 and 12.9%) during repeated, leg extension muscle actions to failure.

## 5 Conclusion

In the present study there was an acute decrease in the ability to remain vigilant and respond correctly to visual stimuli as indicated by the increased PVT_RT_ during MH (FiO_2_: 15.4%) and increased PVT_RT_ and PVT_E_ during SH (FiO_2_: 12.9%) conditions. Furthermore, it is likely that acute hypoxic exposure while performing short duration, high intensity exercise was not sufficient stimuli to elicit hypoxic-specific changes in HR responses, muscular strength (1-RM), or repetitions to failure. The SpO_2_ responses were hypoxic-level dependent with increasing levels of hypoxia resulting in greater and more sustained reductions in SpO_2_. The combined SpO_2_ and StO_2_ responses at MH and SH suggested a balance between the muscles metabolic demand remaining lower than the muscle oxygen diffusion capacity. During the SH condition, the localized muscle recovery Abs-StO_2_ likely experienced greater metabolic stress than Normoxia and MH conditions during the fatiguing leg extension tasks resulting in lower muscle oxygenation levels indicating a greater systemic prioritization placed on oxygenation. The patterns of responses for StO_2_% during the three sets of leg press to failure indicated that exercise is a more potent influencer to muscle oxygenation status than hypoxic conditions (FiO_2_: 15.4 and 12.9%) during repeated, leg extension muscle actions to failure.

## Funding

There was no funding for this study.

## CRediT authorship contribution statement

**Cory M. Smith:** Conceptualization, Methodology, Software, Formal analysis, Investigation, Data curation, Writing – original draft, Writing – review & editing, Visualization. **Owen F. Salmon:** Conceptualization, Methodology, Software, Investigation, Data curation, Writing – review & editing, Visualization. **Jasmin R. Jenkins:** Conceptualization, Methodology, Software, Investigation, Data curation, Writing – review & editing, Visualization.

## Declaration of competing interest

The authors declare that they have no known competing financial interests or personal relationships that could have appeared to influence the work reported in this paper.

## References

[bib1] Alvar B., Sell K., Deuster P. (2017). Essentials of Tactical Strength and Conditioning.

[bib2] Attias J., Bieles J., Carvil P., Laing C., Lewis F., Jaka O., O'Brien K., Ruchaya P. (2017). Altitude exposure and increased heart rate: the role of the parasympathetic nervous system. J. Physiol..

[bib3] Baechle T.R., Earle R.W., Strength N., Association C. (2008). Human Kinetics.

[bib4] Basner M., Dinges D.F. (2012). An adaptive-duration version of the PVT accurately tracks changes in psychomotor vigilance induced by sleep restriction. Sleep.

[bib5] Bishop P.A., Fielitz L.R., Crowder T.A., Anderson C.L., Smith J.H., Derrick K.R. (1999). Physiological determinants of performance on an indoor military obstacle course test. Mil. Med..

[bib6] Bourdillon N., Mollard P., Letournel M., Beaudry M., Richalet J.-P. (2009). Interaction between hypoxia and training on NIRS signal during exercise: contribution of a mathematical model. Respir. Physiol. Neurobiol..

[bib7] Davis M.L., Barstow T.J. (2013). Estimated contribution of hemoglobin and myoglobin to near infrared spectroscopy. Respir. Physiol. Neurobiol..

[bib8] Decamp E., Schneider J.S. (2009). Interaction between nicotinic and dopaminergic therapies on cognition in a chronic Parkinson model. Brain Res..

[bib9] Guo J.-Y., Zheng Y.-P., Xie H.-B., Chen X. (2010). Continuous monitoring of electromyography (EMG), mechanomyography (MMG), sonomyography (SMG) and torque output during ramp and step isometric contractions. Med. Eng. Phys..

[bib10] Hill N.E., Stacey M.J., Woods D. (2011). Energy at high altitude. J. Roy. Army Med. Corps.

[bib11] Jones S., Chiesa S.T., Chaturvedi N., Hughes A.D. (2016). Recent developments in near-infrared spectroscopy (NIRS) for the assessment of local skeletal muscle microvascular function and capacity to utilise oxygen. Artery Research.

[bib12] Khitrov M.Y., Laxminarayan S., Thorsley D., Ramakrishnan S., Rajaraman S., Wesensten N.J., Reifman J. (2014). PC-PVT: a platform for psychomotor vigilance task testing, analysis, and prediction. Behav. Res. Methods.

[bib13] Koller E.A., Drechsel S., Hess T., Macherel P., Boutellier U. (1988). Effects of atropine and propranolol on the respiratory, circulatory, and ECG responses to high altitude in man. Eur. J. Appl. Physiol. Occup. Physiol..

[bib14] Kumar A., Ownby R., Waldrop-Valverde D., Fernandez B., Kumar M. (2011). Human immunodeficiency virus infection in the CNS and decreased dopamine availability: relationship with neuropsychological performance. J. Neurovirol..

[bib15] Latshang T.D., Lo Cascio C.M., Stöwhas A.-C., Grimm M., Stadelmann K., Tesler N., Achermann P., Huber R., Kohler M., Bloch K.E. (2013). Are nocturnal breathing, sleep, and cognitive performance impaired at moderate altitude (1,630–2,590 m)?. Sleep.

[bib16] Lenhart M. (2012).

[bib17] Lucía A., Sánchez O., Carvajal A., Chicharro J.L. (1999). Analysis of the aerobic-anaerobic transition in elite cyclists during incremental exercise with the use of electromyography. Br. J. Sports Med..

[bib18] Matsangas P., Shattuck N.L., Brown S. (2017). Preliminary validation study of the 3-min wrist-worn psychomotor vigilance test. Behav. Res. Methods.

[bib19] Mazzeo R.S. (2008). Physiological responses to exercise at altitude. Sports Med..

[bib20] McManus C.J., Collison J., Cooper C.E. (2018). Performance comparison of the MOXY and PortaMon near-infrared spectroscopy muscle oximeters at rest and during exercise. J. Biomed. Opt..

[bib21] McMorris T., Hale B.J., Barwood M., Costello J., Corbett J. (2017). Effect of acute hypoxia on cognition: a systematic review and meta-regression analysis. Neurosci. Biobehav. Rev..

[bib22] Millet G.Y., Aubert D., Favier F.B., Busso T., Benoît H. (2009). Effect of acute hypoxia on central fatigue during repeated isometric leg contractions. Scand. J. Med. Sci. Sports.

[bib23] Millet G.Y., Muthalib M., Jubeau M., Laursen P.B., Nosaka K. (2012). Severe hypoxia affects exercise performance independently of afferent feedback and peripheral fatigue. J. Appl. Physiol..

[bib24] Oeung B., Heinrich E.C., Puvvula N., Pham K., Frost S., Brena R., DeYoung P.N., Orr J.E., Malhotra A. (2020). Effects of acclimatization on cognitive function at high altitude. Faseb. J..

[bib25] Ogoh S., Ainslie P.N. (2009). Cerebral blood flow during exercise: mechanisms of regulation. J. Appl. Physiol..

[bib26] Pun M., Hartmann S.E., Furian M., Dyck A.M., Muralt L., Lichtblau M., Bader P.R., Rawling J.M., Ulrich S., Bloch K.E., Poulin M.J. (2018). Effect of acute, subacute, and repeated exposure to high altitude (5050 m) on psychomotor vigilance. Front. Physiol..

[bib27] Rajaraman S., Ramakrishnan S., Thorsley D., Wesensten N.J., Balkin T.J., Reifman J. (2012). A new metric for quantifying performance impairment on the psychomotor vigilance test. J. Sleep Res..

[bib28] Sander M., Roach R.C., Hackett P.H., Wagner P.D. (2016). Hypoxia: Translation in Progress.

[bib29] Scofield D.E., Kardouni J.R. (2015). The tactical athlete: a product of 21st century strength and conditioning. Strength Condit. J..

[bib30] Shannon O.M., Duckworth L., Barlow M.J., Deighton K., Matu J., Williams E.L., Woods D., Xie L., Stephan B.C.M., Siervo M., O'Hara J.P. (2017). Effects of dietary nitrate supplementation on physiological responses, cognitive function, and exercise performance at moderate and very-high simulated altitude. Front. Physiol..

[bib31] Shaw D.M., Cabre G., Gant N. (2021). Hypoxic hypoxia and brain function in military aviation: basic physiology and applied perspectives. Front. Physiol..

[bib32] Shibuya K., Tanaka J. (2003). Skeletal muscle oxygenation during incremental exercise. Arch. Physiol. Biochem..

[bib33] Shukitt-Hale B., Banderet L.E., Lieberman H.R. (1998). Elevation-dependent symptom, mood, and performance changes produced by exposure to hypobaric hypoxia. Int. J. Aviat. Psychol..

[bib34] Spires J., Lai N., Zhou H., Saidel G.M., LaManna J.C., Puchowicz M.A., Xu K., Harrison D.K., Bruley D.F. (2011). Oxygen Transport to Tissue XXXII.

[bib35] Taylor A.D., Bronks R. (1994). Electromyographic correlates of the transition from aerobic to anaerobic metabolism in treadmill running. Eur. J. Appl. Physiol. Occup. Physiol..

[bib36] WMA (2013). World Medical Association Declaration of Helsinki: Ethical principles for medical research involving human subjects. Jama.

